# Viral etiology of life‐threatening pediatric pneumonia: A matched case‐control study

**DOI:** 10.1111/irv.12738

**Published:** 2020-04-08

**Authors:** Jianxing Yu, Suyun Qian, Chunyan Liu, Yan Xiao, Teng Xu, Ying Wang, Hang Su, Lan Chen, Bin Yuan, Xinming Wang, Baoping Xu, Yan Yang, Kunling Shen, Zhengde Xie, Lili Ren, Jianwei Wang

**Affiliations:** ^1^ National Health Commission Key Laboratory of Systems Biology of Pathogens and Christophe Merieux Laboratory Institute of Pathogen Biology Chinese Academy of Medical Sciences & Peking Union Medical College Beijing China; ^2^ Beijing Key Laboratory of Pediatric Respiratory Infection Diseases Key Laboratory of Major Diseases in Children Ministry of Education National Clinical Research Center for Respiratory Diseases National Key Discipline of Pediatrics (Capital Medical University) Beijing Pediatric Research Institute Beijing Children’s Hospital Capital Medical University National Center for Children’s Health Beijing China; ^3^ Vision Medicals Guangzhou China; ^4^ The University of Texas Health Science Center at San Antonio San Antonio TX USA

**Keywords:** case‐control study, children, critical care, influenza A virus, pneumonia, respiratory virus

## Abstract

**Background:**

Pediatric pneumonia remains a significant health challenge, while the viral risk factors for adverse outcomes in pediatric pneumonia are not yet fully clear.

**Methods:**

A matched case‐control study of pediatric patients with pneumonia was carried out in Beijing, China, between 2007 and 2015. The study enrolled 334 intensive care unit patients who developed life‐threatening diseases and 522 controls matched to the sex, age, ethnicity, admission dates, and residing district of the cases suffered from pneumonia. Nasopharyngeal aspirates were taken from all participants and tested by PCR for 18 common respiratory viruses.

**Results:**

At least, one virus was detected in 257 (77%) of the cases and 409 (78%) of the controls. We observed no difference in the prevalence of 17 respiratory viruses between cases and controls but found a higher frequency of influenza A virus (IFV‐A) in the cases than in the controls (7% vs 4%, *P* = .036). After adjusting for comorbid conditions and a history of reactive airway diseases, IFV‐A was associated with an increase in life‐threatening pneumonia (adjusted odds ratio = 2.55, 95% CI = 1.24‐5.24). Young age and congenital heart disease (aOR = 10.16‐10.27, *P* < .001) were also independent risk factors.

**Conclusions:**

The prevention of IFV infection is critical in decreasing the risk of life‐threatening pneumonia in children.

## 1 INTRODUCTION

Pneumonia is a leading cause of death globally, particularly for children._1_ One in five children worldwide suffers an episode of pneumonia each year,_2_ and 12%‐25% of pediatric pneumonia patients progress to severe pneumonia requiring hospital referral and treatment. Roughly 21% of children who require hospitalization for pneumonia are admitted into a pediatric intensive care unit (PICU), resulting in death in as many as 1% of cases._3_, _4_ Due to the health burden of pediatric pneumonia, there is a growing interest in clarifying the etiological agents that contribute to the severity and progression of the disease, and respiratory viruses have been explored in this context.

The respiratory viruses associated with the incidence of pneumonia have been reported._5_, _6_, _7_ However, few data described the contributions of viral agents to the development of severe pneumonia among children in inpatient settings. The understanding of the risk factors that predispose pediatric pneumonia patients to life‐threatening events may help improve clinical care, guide diagnostic testing, reduce the number of unnecessary antibiotic prescriptions, decrease medical costs, and lower the risk of nosocomial infections in a hospital setting.^8^

To clarify the contributions of respiratory viruses to very severe pediatric pneumonia, we conducted a matched case‐control study to assess the contributions of several common respiratory viruses.

## 2 MATERIALS AND METHODS

### 2.1 Study design

A matched case‐control study was designed to analyze a cohort of pediatric patients with pneumonia at Beijing Children's Hospital from April 1, 2007, to December 31, 2015, in Beijing, China. The following three criteria were used to enroll patients: (a) had symptoms of acute infection, including fever (body temperature >38.0°C) or hypothermia (body temperature <35.5°C), leukocytosis (for children <5 years old, white blood cell count [WBC] >15 000/mL, and for children ≥5 years old, WBC > 11 000/mL), or leukopenia (for children <5 years old, WBC < 5000/mL, and for children ≥5 years old, WBC < 4000/mL); (b) had signs and/or symptoms of lower respiratory tract infections, including cough, sputum production, shortness of breath, tachypnea (based on age; <2 months, ≥60 breaths/min; 2‐11 months, ≥50 breaths/min; 12‐59 months, ≥40 breaths/min; >5 years, ≥30 breaths/min), wheezing or crackles, dyspnea, or chest pain; and (c) had radiograph evidence suggestive of pneumonia or pleural effusion. Patients were excluded if they were younger than 28 days or older than 14 years of age, with known immunosuppressive diseases including solid organ or hematopoietic stem cell transplants, receiving chemotherapy, HIV‐positive or had AIDS, and taking steroids for more than 30 days. To avoid duplication, patients who had recurrent hospitalizations within 3 months of enrollment were also excluded.

Nasopharyngeal aspirates were collected from each enrolled patient. Nucleic acids were extracted from the aspirates and screened for the presence of 18 respiratory viruses using reverse transcriptase‐polymerase chain reaction (RT‐PCR) and PCR assays as described previously._9_ The viral agents screened included influenza viruses (IFV‐A, ‐B, and ‐C), human rhinovirus (HRV), human parainfluenza virus 1‐4 (HPIV‐1, ‐2, ‐3, and ‐4), respiratory syncytial virus (RSV‐A and ‐B), human adenovirus (Adv), enterovirus (EV), human bocavirus (HBoV), human metapneumovirus (HMPV), and human coronavirus (HCoV‐229E, OC43, NL63, and HKU1). We did not perform IFV‐A subtyping assays in the study.

All hospitalized patients were reviewed retrospectively to determine the assignment of cases and controls. All patients who were admitted into the PICU and developed life‐threatening diseases, defined as mechanical ventilation, sepsis, shock, or death, were defined as cases. These patients ranged in ages from 28 days to 13 years. Control patients were selected from general wards and did not present with life‐threatening diseases. For each case, up to two controls were matched according to sex, age (±2 months), ethnicity, admission date (±15 days), and residing district. If no eligible controls were found, the matched conditions were expanded to include patient ±6 months of age and with an admission date of ±4 weeks for patients <5 years of age, or age of ±1 years and an admission date of ±4 weeks in patients more than 5 years of age. Two controls were selected at random if there were more than two eligible controls in the cohort.

To validate the data, two pediatricians and one pediatric radiologist independently reviewed all chest radiographs, important clinical outcomes, and diagnoses. Inconsistent diagnoses were discussed with a third senior pediatrician, who would make a final decision. Clinical outcomes or diagnoses used to define the cases and controls were collected from medical charts at the time of hospital discharge, including PICU admission, non‐invasive ventilation, for example, continuous positive airway pressure (CPAP), invasive ventilation including mechanical ventilation involving tracheostomy or endotracheal tube, acute respiratory failure (code 518.81) according to the International Classification of Diseases, ninth revision (ICD‐9), shock (ICD‐9 code 785.5x), sepsis (ICD‐9 code 038.xx), and death. Baseline data at hospital admission, including demographic data (sex and age), epidemiological data (date of illness onset and prematurity history of children defined as gestational age of children <37 weeks), and clinical data (symptoms/signs and comorbid conditions) were retrieved from study records. Observed comorbid conditions included congenital heart disease (CHD), defined as children with a diagnosis ICD‐9 coded as 745.xx, 746.xx, and 747.xx, chronic lung disease, chromosomal anomalies, moderate‐to‐severe anemia (haemoglobin <90 g/L), and malnutrition.

The study was approved by the ethical review committee at the Institute of Pathogen Biology, Chinese Academy of Medical Sciences (IPB, Beijing, China [ref. IPB‐2014‐4]). Written consent was obtained before enrollment from each patient or their guardians.

### 2.2 Statistical analysis

We used chi‐square tests or Fisher's exact tests to analyze categorical variables and Wilcoxon rank‐sum tests to analyze continuous variables as appropriate. A two‐sided *P*‐value of < .05 was considered statistically significant. Conditional logistic regression was used to explore the relationship between viral exposure at hospital admission and subsequent life‐threatening disease during hospitalization. The comorbidities, breast‐feeding, influenza vaccination last year, and initial antibiotic usage were also included in the multivariable conditional logistic regression to account for potential confounders if they were significant at *P* < .10 in univariate analysis. Data are reported as crude or adjusted odds ratio (OR) with a 95% confidence interval (95% CI). We also conducted sensitive analysis between different age strata, for example, children aged 1‐59 months and children 5‐13 years. All analyses were conducted in r version 2.15.3 (R Foundation for Statistical Computing).^10^

## 3 RESULTS

### 3.1 Characteristics of subjects

**Figure 1:**
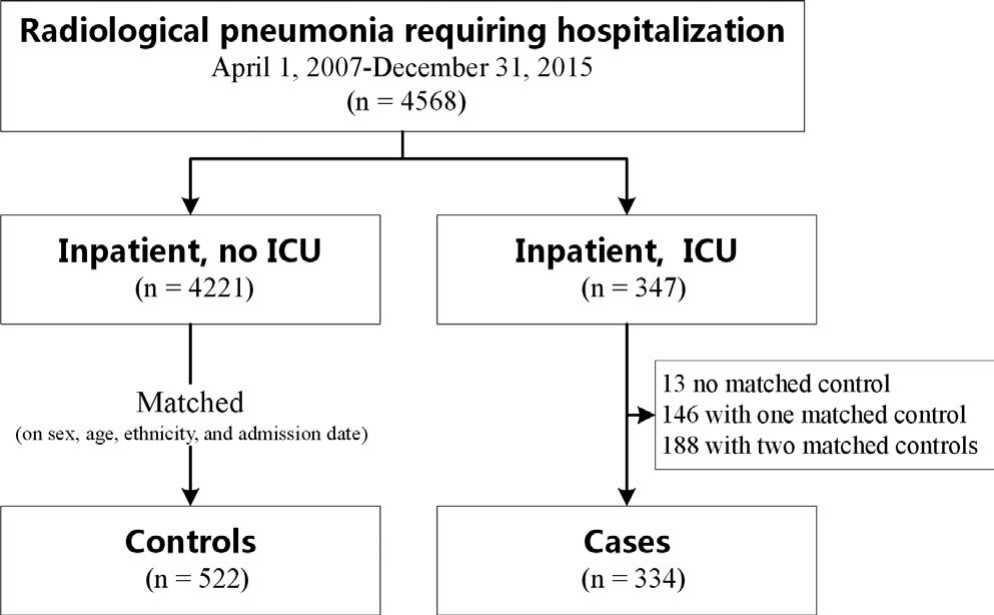
Flow diagram of case and control selection process. Abbreviations: ICU, intensive care unit

During the study period, a total of 4568 pediatric patients were hospitalized for pneumonia. We identified 347 (8%) PICU admissions with life‐threatening events during hospitalization, of which 13 cases were excluded because no eligible matched controls were available, even using the expanding matched criteria (Figure 1). A total of 334 cases and 522 matched controls were included in our analysis, for which 188 (56%) had two matched controls and 146 (44%) had one matched control. Demographic and vaccination information as well as descriptions of the underlying disease are provided in Table 1. The median age of the cases was 6 months, and 49% (n = 162) of PICU admissions were for children younger than 6 months. The frequency of congenital heart diseases was higher in the cases than in the controls (38% vs 8%, *P* < .001, Fisher's exact test). The rate of antibiotic usage (93% vs 99%, *P* < .001, Fisher's exact test) and the prevalence of a history of asthma and other reactive airway diseases (2% vs 5%, *P* < .024, Fisher's exact test) were lower in the cases than in the controls. The rate of influenza vaccination in the past year was not statistically different in the cases and the controls (14% vs 14%, *P* = .841, Fisher's exact test) (Table 1). All the patients in both group had no history of anti‐viral drugs treatment before seeing a doctor.

**Table 1:** Demographic and clinical characteristics of study subjects

Characteristic	Cases (n = 334)	Controls (n = 522)	*P*‐value
Median age in months (IQR)	6 (2‐16)	6 (3‐19)	.336
Age in months			.054
1 mo	62 (19)	62 (12)	
2‐5 mo	100 (30)	189 (36)	
6‐11 mo	62 (19)	84 (16)	
12‐23 mo	45 (13)	70 (13)	
24‐59 mo	38 (11)	61 (12)	
60 + mo	27 (8)	56 (11)	
Male sex	218 (65)	372 (71)	.069
History of prematurity	27 (8)	39 (7)	.793
Breast‐feeding	70 (21)	121 (23)	.501
History of past respiratory illness	74 (22)	101 (19)	.340
Pneumonia >3 mo ago	60 (18)	74 (14)	.148
Asthma and other reactive airway diseases	6 (2)	25 (5)	***.024***
Measles, tuberculosis, and other infectious disease	11 (3)	12 (2)	.393
Comorbid conditions	157 (47)	88 (17)	***<.001***
Congenital heart disease	125 (38)	41 (8)	***<.001***
Cyanotic congenital heart defects^a^	13 (4)	3 (1)	***<.001***
Acyanotic congenital heart defects	112 (34)	38 (7)	***<.001***
Congenital air way or chromosomal anomalies	32 (10)	34 (7)	.115
Moderate‐to‐severe anemia	14 (4)	11 (2)	.096
Chronic lung diseases	2 (1)	5 (1)	.711
Malnutrition	2 (1)	2 (0)	.646
Influenza vaccination last year	46 (14)	75 (14)	.841
Ongoing antibiotic treatment	312 (93)	516 (99)	***<.001***

Note

Data are presented as no. (%) of patients unless otherwise indicated.

Abbreviation: IQR, interquartile range.

^a^ Congenital heart disease (defined as International Classification of Diseases, Ninth Revision, codes 745.xx, 746.xx, or 747.xx) was diagnosed at hospital admission, and cyanotic congenital heart defects included Fallot's tetralogy (ICD‐9 code 745.2), transposition of the great arteries (ICD‐9 code 745.1x), or tricuspid atresia (ICD‐9 code 746.1).

### 3.2 Association between respiratory viruses and life‐threatening events

**Figure 2:**
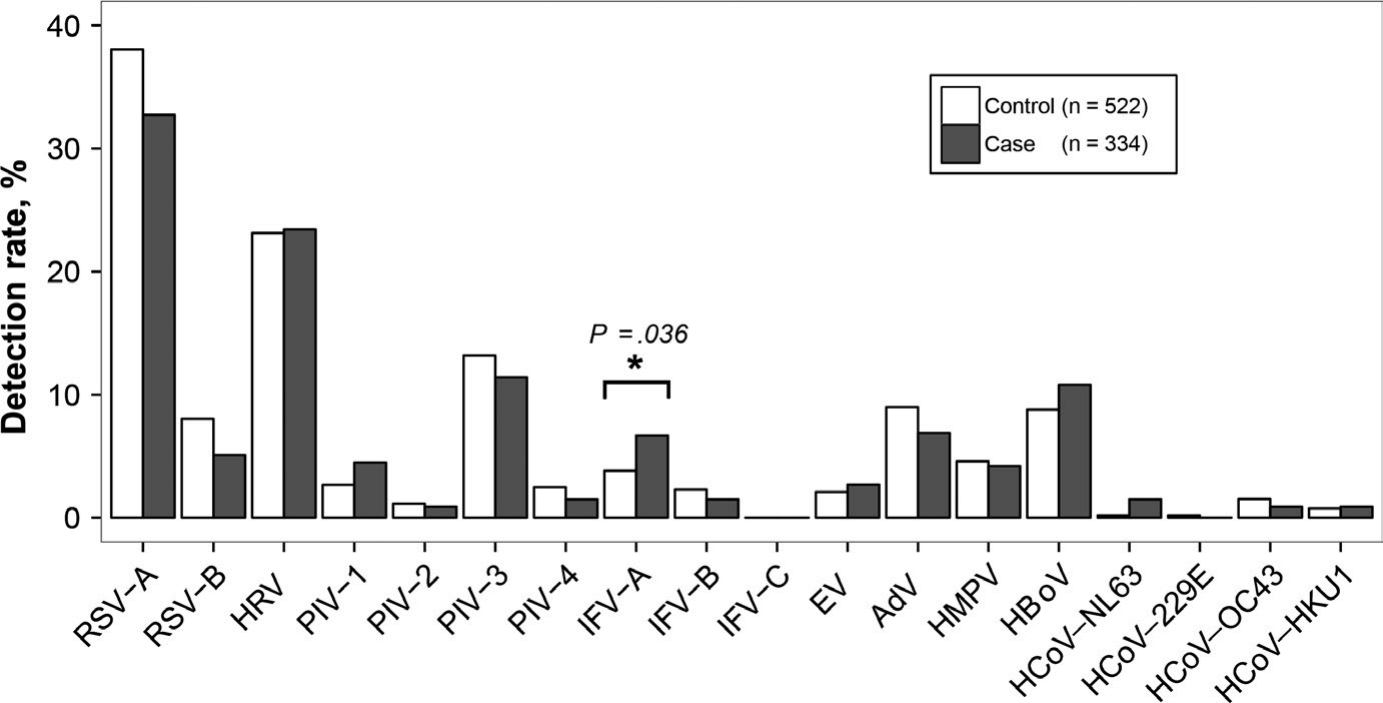
Frequency of respiratory viruses detected in cases and controls. Abbreviations: Adv, human adenovirus; EV, enterovirus; HBoV, human bocavirus; HCoV, human coronavirus; HMPV, human metapneumovirus; HRV, human rhinovirus; IFV, influenza virus; PIV, human parainfluenza virus; RSV, respiratory syncytial virus

At least, one virus was detected in 257 (77%) cases and in 409 (78%) controls. The species and subtypes of respiratory virus detected in the cases and controls are listed in Table 2. In both the cases and the controls, the detection rates of the respiratory viruses were RSV (38%, 46%), HRV (23%, 23%), HPIV (17%, 19%), HBoV (11%, 9%), Adv (7%, 9%), hMPV (4%, 5%), HCoV (4%, 3%), and EVs (3%, 2%) (Figure 2). Among all the detected viruses, only the frequency of IFV‐A virus was greater in the cases than in the controls (7% vs 4%, *P* = .036, Fisher's exact test), while the other respiratory viruses showed no significant difference between the two groups.

**Table 2:** Frequency of tested respiratory viruses in cases and controls

Respiratory viruses	Case (n = 334)	Control (n = 522)	Unadjusted ORs (95% CI)	Adjusted ORs (95% CI)
Influenza virus	28 (8)	31 (6)	1.59 (0.89‐2.84)	
A	23 (7)	19 (4)	***2.55 (1.24‐5.24)***	***2.52 (1.15‐5.55)***
B	5 (2)	12 (2)	0.57 (0.19‐1.68)	
C	0 (0)	0 (0)	‐	
Human rhinovirus	78 (23)	121 (23)	0.98 (0.69‐1.39)	
Parainfluenza virus	56 (17)	97 (19)	0.86 (0.59‐1.26)	
Type‐1	15 (5)	14 (3)	1.54 (0.73‐3.22)	
Type‐2	3 (1)	6 (1)	0.62 (0.15‐2.51)	
Type‐3	38 (11)	69 (13)	0.85 (0.54‐1.33)	
Type‐4	5 (2)	13 (2)	0.56 (0.19‐1.62)	
Enterovirus	9 (3)	11 (2)	1.51 (0.60‐3.76)	
Human adenovirus	24 (7)	46 (9)	0.81 (0.46‐1.41)	
Human coronavirus	14 (4)	18 (3)	1.24 (0.59‐2.57)	
NL63	5 (2)	1 (0)	6.53 (0.75‐56.92)	
229E	0 (0)	1 (0)	‐	
OC43	3 (1)	8 (2)	0.65 (0.17‐2.48)	
HKU1	3 (1)	4 (1)	1.11 (0.24‐5.05)	
Respiratory syncytial virus	127 (38)	239 (46)	***0.68 (0.48‐0.94)***	0.75 (0.51‐1.10)
Subgroup A	109 (33)	199 (38)	0.72 (0.51‐1.03)	
Subgroup B	18 (5)	41 (8)	0.71 (0.39‐1.27)	
Human metapneumovirus	14 (4)	24 (5)	0.93 (0.45‐1.95)	
Human bocavirus	37 (11)	45 (9)	1.34 (0.83‐2.17)	
Positive any virus	257 (77)	409 (78)	0.94 (0.65‐1.37)	
Single detected virus	160 (48)	234 (45)	1.16 (0.87‐1.54)	
Multiple detected viruses	97 (29)	175 (34)	0.80 (0.58‐1.10)	
Two pathogens	70 (21)	134 (26)	0.76 (0.55‐1.07)	
Three pathogens	22 (7)	33 (6)	1.06 (0.59‐1.89)	
≥Four pathogens	5 (1)	8 (2)	0.94 (0.65‐1.37)	

Note

Data are presented as no. (%) of patients unless otherwise indicated.

To clarify the contributions of viruses to life‐threatening disease, we analyzed the associations between each virus and an adverse clinical record. IFV‐A was analyzed firstly as its higher frequency in the cases. Univariate analysis revealed that IFV‐A was significantly associated with an increased frequency of life‐threatening events (OR = 2.55, 95% CI = 1.24‐5.24). This association persisted (adjusted odds ratio (aOR) =2.52, 95% CI = 1.15‐5.55) after adjusting for potential confounders, including comorbidities, antibiotic usage, and a history of other reactive airway diseases. Although RSV was the most detected viruses in the two groups, however, RSV‐positive detection was negatively associated with the life‐threatening events (OR = 0.68, 95% CI = 0.48‐0.94). After controlling for confounding variables, we did not observe an association between RSV and the occurrence of life‐threatening events (aOR = 0.75, 95% CI = 0.51‐1.10). Sensitive analysis showed similar results in children aged 1‐59 months (Table S1). In adjusted models for RSV infection or IFV‐A infection, congenital heart disease (aOR = 10.16 [95% CI = 5.94‐17.37] for RSV and aOR = 10.27 [95% CI = 6.06‐17.75] for IFV‐A) was strongly associated with an increased frequency of life‐threatening pneumonia.

We also analyzed the codetection rates of respiratory viruses, but observed no significant difference in the cases (97, 29%) and controls (175, 34%) (*P* = .241, Fisher's exact test) (Table 2). RSV with HRV were in highest codetection frequency in both cases (n = 24, 7%) and controls (n = 45, 9%).

### 3.3 Clinical signs and outcomes in relation to viral findings

We then analyzed the relationships between clinical symptoms, prognosis, and the tested respiratory viruses. Although only 42 children were IFV‐A‐positive, these patients had higher rates of pediatric PICU admission (55% vs 38%, *P* = .036), invasive ventilation (36% vs 10%, *P* < .001), and acute respiratory failure (45% vs 29%, *P* = .024) than did the IFV‐A negative patients with Fisher's exact test. Fever > 38.5°C (63% vs 40%, *P* = .005) and tachycardia (60% vs 41%, *P* = .024) were positively associated with IFV‐A positive detections with Fisher's exact test (Table 3).

**Table 3:** Comparison of clinical characteristics in children suffered from pneumonia with Influenza A virus positive or negative detections

Characteristic	Positive (n = 42)	Negative ( n = 814)	*P*‐value
Clinical signs			
Leukocytosis	23 (55)	389 (48)	.430
Leukopenia	2 (5)	22 (3)	.331
Fever (body temperature >38.0°C)	32 (76)	471 (58)	.023
Hypothermia (body temperature <35.5°C)	0 (0)	5 (1)	1
Tachypnea	14 (33)	175 (21)	.085
Tachycardia	25 (60)	334 (41)	***.024***
Chest indrawing	21 (50)	345 (42)	.341
Persistent vomiting	3 (7)	37 (5)	.440
Treatment			
Antibiotics	42 (100)	786 (97)	***<.001***
Anti‐virals	10 (24)	204 (25)	1
Bronchodilators	8 (19)	83 (10)	.075
Steroids	38 (90)	650 (80)	.110
Clinical outcomes			
LoS, median days, (IQR)	13 (9‐17)	11 (8‐16)	.271
Admission to pediatric ICU	23 (55)	311 (38)	***.036***
Transferred immediately to pediatric ICU upon admission	18 (43)	259 (32)	.1749
Mechanical ventilation	23 (55)	298 (37)	***.022***
Invasive ventilation	15 (36)	81 (10)	***<.001***
Tracheostomy	5 (12)	21 (3)	***.007***
Endotracheal tube	14 (33)	74 (9)	***<.001***
Non‐invasive, for example, CPAP	18 (43)	275 (34)	.245
Respiratory failure	19 (45)	232 (29)	***.024***
Shock	1 (2)	13 (2)	.508
Sepsis	1 (2)	31 (4)	1
Death	0 (0)	8 (1)	1

Note

Data are presented as no. and (%) of patients unless otherwise indicated.

Abbreviations: CPAP, continuous positive airway pressure; ICU, intensive care unit; IQR, interquartile range; LoS, length of hospital stay.

RSV was the most frequently detected virus in hospitalized patients, but RSV‐positive patients were less likely to develop life‐threatening disease, as evidenced by lower rates of pediatric PICU admission (35% vs 42%, *P* = .028), invasive ventilation (7% vs 14%, *P* < .001), shock (0% vs 3%, *P* = .006), sepsis (2% vs 5%, *P* = .045), and death (0% vs 2%, *P* = .012), compared to children tested negative for RSV with Fisher's exact test. Fever > 38.5°C (26% vs 52%, *P* < .001) was observed less frequently in RSV‐positive patients, but chest indrawing (53% vs 35%, *P* < .001) was more frequent in RSV‐positive patients compared to RSV‐negative subjects (Table S2).

At hospital discharge, 153 (18%) children were considered cured of respiratory disease, 624 (73%) had achieved clinical stability, 8 (1%) were deceased, and 71 (8%) gave up treatment and left the hospital due to disease progression or personal reasons. All eight deaths occurred in the pediatric PICU; two of these patients were positive for Adv, one was positive for EV, one was positive for both HPIV‐1 and HBoV, and four were negative for all tested respiratory viruses.

## 4 DISCUSSION

Pediatric patients suffering from pneumonia are disproportionately affected by etiologies at different stage of disease development._1_, _11_ At the community level, a majority of episodes of pneumonia in children are caused by respiratory viruses, with RSV (~29% of all pneumonia) and IFV (17%) being the most common viral causes._1_, _5_ In contrast, 56%‐78% of severe pneumonia is associated with respiratory viruses at the severe pneumonia level, and RSV is considered contributing decreased to 23% (range: 18%‐34%) and IFVs to 7%._1_, _3_, _12_, _13_, _14_, _15_, _16_ At the third level, child deaths caused by viral pathogens are low among children with pneumonia, with only 6.6% of deaths being attributed to RSV and IFVs._17_ It is well‐known that most mild‐to‐moderate childhood pneumonia is caused by viruses, whereas most severe and fatal pneumonia cases are caused by bacterial infections._11_, _17_ Although the prevalence of RSV is high in hospitalized children in previous study,_3_ deaths associated with RSV are uncommon in high‐resource settings, and the majority of these deaths occur in children with chronic conditions.^18^, ^19^

In this study, we investigated whether several viral agents are associated with life‐threatening events in hospitalized patients with pneumonia using a case‐control study. We found that more than 80% of enrolled hospitalized patients tested positive for respiratory viruses. As expected, RSV was the most frequently observed virus,_3_, _13_ but RSV showed no association with life‐threatening events in our study. RSV is a major cause of deaths among children with lower respiratory tract illnesses living in low‐income to low–middle‐income countries, responsible for ~ 60 000 deaths annually in developing countries in 2015._20_ The gross regional product per capita in Beijing in 2015 was 106 497 Chinese Yuan ($17 098),_21_ which belongs to the high‐income regions but not that of low‐income to low–middle‐income regions according to the World Bank's classification of economies (gross national income per capita ≥ $12 376)._22_ After adjusting for confounding factors, IFV‐A was the only viral agent associated with life‐threatening pneumonia in children requiring hospitalization, and it was associated with a two‐fold increase in life‐threatening events. Although IFV‐A was identified less frequently in hospitalized patients, 36% of IFV‐A‐positive inpatients received invasive ventilation and 45% progressed to acute respiratory failure. These results suggest that IFV‐A is an important etiological pathogen in the context of pediatric critical care. IFV infection has previously been reported to be associated with an increased risk of cardiac events in adults and with adverse outcomes (eg, shock, neurological disease, organ failures, and death) in children._23_, _24_ Current clinical guidelines for the management of childhood pneumonia strongly recommend rapid tests for IFV in an inpatient setting._25_, _26_ Our results further emphasize the critical role of IFV‐A infections in severe pneumonia in hospitalized pediatric patients. Early pathogen diagnostics and timely anti‐viral therapies would improve mortality.^25^, ^26^

We also analyzed the relationships between several host factors and life‐threatening events. In our study, almost half of children who had life‐threatening pneumonia or who were admitted into the PICU were younger than 6 months of age, and 47% had a pre‐existing comorbid condition at the time of hospital admission. Congenital heart disease was the most common comorbidity in children with life‐threatening pneumonia in critical care (38%), associated with a 10‐fold increase in life‐threatening events, regardless of viral infections. Young age and congenital heart disease have previously been reported as risk factors for PICU admission using cohorts suffering from severe pneumonia._15_ In Hall et al’s study on children with acute respiratory infections, young age and prematurity were risk factors for hospitalization with RSV infection._14_ Our results showed that young age and congenital heart disease were independent host factors that predispose children to life‐threatening pneumonia. Patients with such host factors on admission should be evaluated promptly and carefully to help improve survival, because delayed admission of patients with severe pneumonia to the PICU is associated with higher mortality as previously reported._27_ The influenza vaccination status of children was not associated with life‐threatening pneumonia in our study. Because the number of children with influenza A virus (n = 42, 5%) was low in our study, the sample size may be underpowered to conclude the effect of vaccination on influenza virus‐associated pneumonia. As we performed a case‐control study, this may lead to our samples less representative of the general population. As about 80% of enrolled patients are <2 years old, asthma and chronic lung disease which are more common in elder children were not often in our study. Cystic fibrosis is also rare in China.

The purpose of this study was to investigate viral etiologies predicting the death or survival of patients with severe pneumonia during hospitalization to optimize site of care. There were only eight recorded cases that resulted in a death during the 8‐year study period, which limits a further analysis. Although an early termination of treatment may indicate an adverse outcome, these patients were not followed up to determine the ultimate clinical outcomes. Instead, we studied life‐threatening events that occurred during hospitalization, including PICU admission, mechanical ventilation, sepsis, shock, and death. In the study, 8% of hospitalized children with pneumonia were transferred to the PICU and experienced at least one the life‐threatening event. The observed proportion of pneumonia patients transferred to the PICU is similar to previous reports studying patients in China,_15_ but is lower than studies conducted in high‐income countries (eg, 21% in the USA),_3_ suggesting that the burden, severity, and risk factors of childhood pneumonia are potentially different between countries._1_, _11_ Policy makers and pediatricians using data from different countries should take into account of these results when optimizing local clinical management strategies and public health policies.

Although our study was conducted in one local children's hospital, however, the results acquired in the study may be generalizable to children living in the other parts of the nation as the prevalence of viruses tested in our study was quite similar to that of other previous studies, confirming the validity of our results. Further multicenter and prospective studies on the association between IFV‐A and life‐threatening pneumonia in children are warranted.

## 5 CONCLUSIONS

This study enriched our understandings on the role of respiratory viruses and host factors in the pneumonia progression of life‐threatening events during hospitalization. Respiratory viruses are a large etiological burden among hospitalized children suffering from pneumonia in China, with RSV being the most common viral agent in children in critical care. The presence of IFV‐A virus is associated with a two‐fold increase in life‐threatening events in pneumonia patients. Rapid etiological diagnostics on admission and specific anti‐viral therapy will be critical for patients receiving critical care. The prevention of IFV‐A virus is recommended in children to reduce the risk of life‐threatening events.

## AUTHOR CONTRIBUTIONS

**Jianxing Yu**: Formal analysis (Lead); Funding acquisition (Supporting); Writing‐original draft‐(Equal); **Suyun Qian**: Data curation (Equal); Investigation (Lead); Resources (Equal); **Chunyan Liu**: Investigation (Equal); Validation (Equal); **Yan Xiao**: Investigation (Equal); Project administration (Equal); Resources (Equal); **Teng Xu**: Software (Equal); Visualization (Equal); **Ying Wang**: Data curation (Equal); Investigation (Equal); Validation (Equal); **Hang Su**: Resources (Equal); Writing‐review & editing (Equal); **Lan Chen**: Investigation (Equal); Project administration (Equal); Validation (Equal); **Bin Yuan**: Software (Equal); Writing‐review & editing (Equal); **Xinming Wang**: Investigation (Equal); Project administration (Equal); Validation (Equal); **Baoping Xu**: Data curation (Equal); Investigation (Equal); Validation (Equal); **Yan Yang**: Data curation (Equal); Investigation (Equal); Resources (Equal); **Kunling Shen**: Supervision (Equal); **Zhengde Xie**: Conceptualization (Equal); Methodology (Equal); Supervision (Equal); Writing‐review & editing (Equal); **Lili Ren**: Conceptualization (Equal); Funding acquisition (Equal); Project administration (Lead); Supervision (Lead); Writing‐review & editing (Equal); **Jianwei Wang**: Conceptualization (Lead); Funding acquisition (Lead); Methodology (Equal).

## Supporting information

Table S1‐S2
