# Supplementary material for: Viral etiology of life‐threatening pediatric pneumonia: A matched case‐control study
Source: Influenza Other Respir Viruses. 2020 Apr 8;14(4):452–9. doi: 10.1111/irv.12738 (PMC7262399; doi:10.1111/irv.12738)
Supplement: Supplementary file 1 — Table S1‐S2 [file IRV-14-452-s001.docx]

Supplementary Table 1. Frequency of tested respiratory viruses in cases and controls, by age groups.

| Respiratory viruses | Children aged 1-59 months | | | |  | Children aged 5-13 years | | | |
| --- | --- | --- | --- | --- | --- | --- | --- | --- | --- |
|  | Case (n=307) | Control (n=466) | Unadjusted ORs (95% CI) | Adjusted ORs (95% CI) |  | Case (n=27) | Control (n=56) | Unadjusted ORs (95% CI) | Adjusted ORs (95% CI) |
| Influenza virus | 26 (8) | 29 (6) | 1.60 (0.83-3.09) |  |  | 2 (7) | 2 (4) | 1.41 (0.08-23.57) |  |
| A | 22 (7) | 18 (4) | ***2.42 (1.05-5.56)*** | ***2.35 (1.10-5.02)*** |  | 1 (4) | 1 (2) | 1.41 (0.08-23.57) |  |
| B | 4 (1) | 11 (2) | 0.67 (0.19-2.35) |  |  | 1 (4) | 1 (2) | 1.00 (0.06-15.99) |  |
| C | 0 (0) | 0 (0) | - |  |  | 0 (0) | 0 (0) | - |  |
| Human rhinovirus | 76 (25) | 118 (25) | 0.85 (0.56-1.28) |  |  | 2 (7) | 3 (5) | 1.41 (0.19-10.34) |  |
| Parainfluenza virus | 55 (18) | 91 (20) | 0.89 (0.58-1.36) |  |  | 1 (4) | 6 (11) | 0.39 (0.04-3.78) |  |
| Type-1 | 15 (5) | 13 (3) | 2.01 (0.87-4.67) |  |  | 0 (0) | 1 (2) | - |  |
| Type-2 | 3 (1) | 4 (1) | 1.05 (0.20-5.61) |  |  | 0 (0) | 2 (4) | - |  |
| Type-3 | 37 (12) | 67 (14) | 0.74 (0.45-1.22) |  |  | 1 (4) | 2 (4) | 1.41 (0.08-23.57) |  |
| Type-4 | 5 (2) | 12 (3) | 0.52 (0.16-1.70) |  |  | 0 (0) | 1 (2) | - |  |
| Enterovirus | 9 (3) | 11 (2) | 1.50 (0.51-4.38) |  |  | 0 (0) | 0 (0) | - |  |
| Human adenovirus | 23 (7) | 45 (10) | 0.85 (0.45-1.61) |  |  | 1 (4) | 1 (2) | 2.00 (0.13-31.98) |  |
| Human coronavirus | 14 (5) | 18 (4) | 1.28 (0.57-2.84) |  |  | 0 (0) | 0 (0) | - |  |
| NL63 | 5 (2) | 1 (0) | 6.53 (0.75-56.92) |  |  | 0 (0) | 0 (0) | - |  |
| 229E | 0 (0) | 1 (0) | - |  |  | 0 (0) | 0 (0) | - |  |
| OC43 | 3 (1) | 8 (2) | 0.75 (0.18-3.08) |  |  | 0 (0) | 0 (0) | - |  |
| HKU1 | 3 (1) | 4 (1) | 0.95 (0.16-5.72) |  |  | 0 (0) | 0 (0) | - |  |
| Respiratory syncytial virus | 127 (41) | 238 (51) | 0.73 (0.50-1.06) |  |  | 0 (0) | 1 (2) | - |  |
| Subgroup A | 109 (36) | 198 (42) | 0.76 (0.50-1.14) |  |  | 0 (0) | 1 (2) | - |  |
| Subgroup B | 18 (6) | 41 (9) | 0.76 (0.40-1.44) |  |  | 0 (0) | 0 (0) | - |  |
| Human metapneumovirus | 14 (5) | 23 (5) | 0.86 (0.37-1.98) |  |  | 0 (0) | 1 (2) | - |  |
| Human bocavirus | 35 (11) | 44 (9) | 1.30 (0.76-2.22) |  |  | 2 (7) | 1 (2) | - |  |

Data are presented as no. (%) of patients unless otherwise indicated.

Supplementary Table 2. Comparison of clinical characteristics in children suffered from pneumonia with respiratory syncytial virus positive or negative detections.

| Characteristic | Positive  (n=366) | Negative  (n=490) | p-value |
| --- | --- | --- | --- |
| Clinical signs |  |  |  |
| Tachypnea | 81 (22) | 108 (22) | 1 |
| Tachycardia | 152 (42) | 207 (42) | 0.889 |
| Chest indrawing | 194 (53) | 172 (35) | <0.001 |
| Persistent vomiting | 11 (3) | 29 (6) | 0.050 |
| Clinical outcomes |  |  |  |
| LoS, median days, (IQR) | 11 (8-15) | 11 (8-17) | 0.147 |
| Admission to pediatric ICU | 127 (35) | 207 (42) | 0.028 |
| Mechanical ventilation | 144 (39) | 177 (36) | 0.354 |
| Invasive ventilation | 25 (7) | 71 (14) | <0.001 |
| Tracheostomy | 5 (1) | 21 (4) | 0.015 |
| Endotracheal tube | 22 (6) | 66 (13) | <0.001 |
| Non-invasive, e.g. CPAP | 137 (37) | 156 (32) | 0.094 |
| Respiratory failure | 114 (31) | 137 (28) | 0.324 |
| Shock | 1 (0) | 13 (3) | 0.006 |
| Sepsis | 8 (2) | 24 (5) | 0.045 |
| Death | 0 (0) | 8 (2) | 0.012 |

Data are presented as no. and (%) of patients unless otherwise indicated. Abbreviations: LoS, Length of hospital stay; ICU, intensive care unit; CPAP, continuous positive airway pressure; IQR, interquartile range.
